# Facile synthesis of transition metal complexes with five coplanar metal-carbon σ-bonds

**DOI:** 10.1093/nsr/nwae078

**Published:** 2024-03-04

**Authors:** Guocheng Jia

**Affiliations:** Department of Chemistry, Hong Kong University of Science and Technology, China

Carbon is one of the most important elements in chemistry, forming a vast number of organic compounds. It can also form a variety of organometallic compounds with a metal–carbon bond. Metal alkyl, carbene and carbyne complexes are classical examples of organometallic compounds. A particularly interesting class of organometallic compounds are all-carbon metallacycles formed by combination of a metal and a hydrocarbon chain serving as a chelating agent with only carbon atoms as the donor atoms. Many interesting monocyclic metallacycles made of a chelating bidentate hydrocarbon chain are known, for example, metallacyclobutadienes, metallacyclopentadienes and metallabenzenes [1]. However, it is challenging to construct polycyclic (or fused) all-carbon metallacycles with a metal at the bridgehead position and a hydrocarbon chain as a polydentate ligand. Reported polycyclic all-carbon metallacycles with three or four metal–carbon σ bonds are still very scarce [2,3], and those with five or more metal–carbon σ bonds remain elusive.

**Figure 1.**
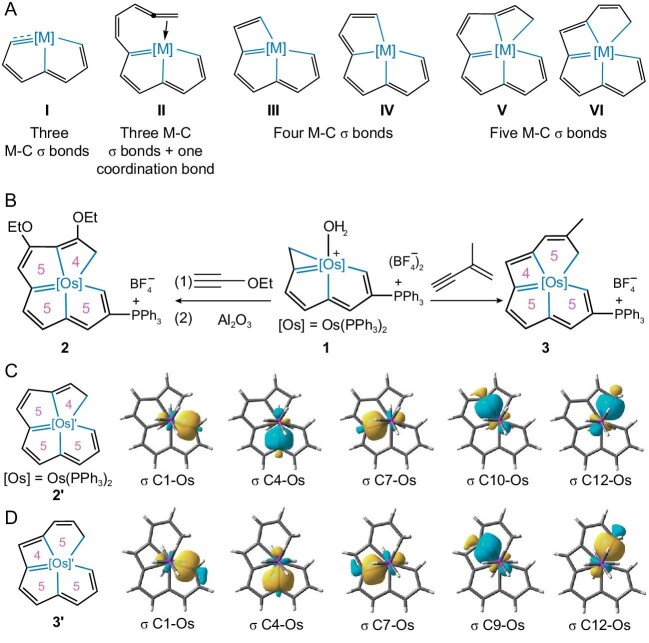
(A) Typical carbolong skeletons. (B) Synthesis of the [5554]-type complex **2** and the [5545]-type complex **3** (the numbers in the rings are the ring sizes). (C and D) Selected Pipek-Mezey localized molecular orbitals (PM-LMO) of **2'** and **3'** in σ-bond forms; isovalue = 0.05. Adapted from Ref. [4].

The team led by Professor Haiping Xia from the Southern University of Science and Technology recently reported an exciting advance in the chemistry of polycyclic all-carbon metallacycles [4]. Professor Xia's group made a breakthrough in metallaaromatics in 2013 by synthesizing and characterizing the first carbolong complexes that contain three coplanar M–C σ bonds (**I**, Fig. 1A) [5]. Subsequently, they have discovered carbolong complexes with other frameworks, including those with three M–C σ bonds and one coordination bond (e.g., **II**, Fig. 1A), and those with four coplanar M–C σ bonds (e.g., **III** and **IV**, Fig. 1A). They have now succeeded in making two novel carbolong complexes containing five coplanar M–C σ bonds (**V** and **VI**, Fig. 1A) [4].

Sequential treatment of complex **1** with ethoxyethyne (HC≡COEt) and excess neutral alumina produced the [5554]-type complex **2**, which contains three five-membered rings and one four-membered ring arranged in the sequence of [5554] (Fig. 1B). Under a similar condition, complex **1** reacted with 2-methyl-1-butene-3-yne (HC≡CCMe=CH_2_) to produce the [5545]-type complex **3**, which also contains three five-membered rings and one four-membered ring, but arranged in the sequence of [5545].

X-ray crystallographic analysis reveals that both complexes adopt a pentagonal bipyramidal geometry with two PPh_3_ ligands occupying the axial positions. The carbon chain acts as a pentadentate ligand with five carbon atoms (four are sp^2^ and one is sp^3^ hybridized) as the donor atoms, forming five coplanar M–C bonds in the equatorial plane. As revealed by density functional theory calculations, the M–C bonds in these complexes have primarily a covalent character. The existence of σ-bonding interactions in the five coplanar M–C bonds are supported by computational work, for example, PM-LMO analysis (Fig. 1C and D). The new complexes are stable at temperatures up to 100°C in moisture or air. The exceptional stability can be attributed to the effect of the conjugated and rigid polydentate systems.

Complexes **2** and **3** are interesting and novel as they represent the first polycyclic carbometallacycles containing five coplanar M–C σ bonds. The work also demonstrated that reactions of alkynes with metallacyclopropenes can be an effective route to fused metallacycles. It is anticipated that the work will serve as an inspiration for new development of carbometallacycles, for example, creation of new structures with five or even more M–C σ bonds or fused rings and new materials with useful properties.
